# Targeting *Mycobacterium tuberculosis* Antigens to Dendritic Cells *via* the DC-Specific-ICAM3-Grabbing-Nonintegrin Receptor Induces Strong T-Helper 1 Immune Responses

**DOI:** 10.3389/fimmu.2018.00471

**Published:** 2018-03-09

**Authors:** Lis Noelia Velasquez, Philipp Stüve, Maria Virginia Gentilini, Maxine Swallow, Judith Bartel, Nils Yngve Lycke, Daniel Barkan, Mariana Martina, Hugo D. Lujan, Hakan Kalay, Yvette van Kooyk, Tim D. Sparwasser, Luciana Berod

**Affiliations:** ^1^Institute of Infection Immunology, TWINCORE, Centre for Experimental and Clinical Infection Research, A Joint Venture between the Medical School Hannover (MHH) and the Helmholtz Centre for Infection Research (HZI), Hannover, Germany; ^2^Mucosal Immunobiology and Vaccine Center (MIVAC), Department of Microbiology and Immunology, Institute of Biomedicine, University of Gothenburg, Gothenburg, Sweden; ^3^Koret School of Veterinary Medicine, Robert H. Smith Faculty of Agriculture, Food and Environment, Hebrew University of Jerusalem, Rehovot, Israel; ^4^Laboratory of Biochemistry and Molecular Biology, School of Medicine, Catholic University of Córdoba, Córdoba, Argentina; ^5^Department of Molecular Cell Biology and Immunology, VU University Medical Center, Amsterdam, Netherlands

**Keywords:** DC-specific-ICAM3-grabbing-nonintegrin, tuberculosis, vaccine, dendritic cells, Ag85B

## Abstract

Tuberculosis remains a major global health problem and efforts to develop a more effective vaccine have been unsuccessful so far. Targeting antigens (Ags) to dendritic cells (DCs) *in vivo* has emerged as a new promising vaccine strategy. In this approach, Ags are delivered directly to DCs *via* antibodies that bind to endocytic cell-surface receptors. Here, we explored DC-specific-ICAM3-grabbing-nonintegrin (DC-SIGN) targeting as a potential vaccine against tuberculosis. For this, we made use of the hSIGN mouse model that expresses human DC-SIGN under the control of the murine CD11c promoter. We show that *in vitro* and *in vivo* delivery of anti-DC-SIGN antibodies conjugated to Ag85B and peptide 25 of Ag85B in combination with anti-CD40, the fungal cell wall component zymosan, and the cholera toxin-derived fusion protein CTA1-DD induces strong Ag-specific CD4^+^ T-cell responses. Improved anti-mycobacterial immunity was accompanied by increased frequencies of Ag-specific IFN-γ^+^ IL-2^+^ TNF-α^+^ polyfunctional CD4^+^ T cells in vaccinated mice compared with controls. Taken together, in this study we provide the proof of concept that the human DC-SIGN receptor can be efficiently exploited for vaccine purposes to promote immunity against mycobacterial infections.

## Introduction

Tuberculosis (Tb) remains one of the leading causes of death worldwide with an estimated 10.4 million people becoming infected per year (1). Currently, the only available vaccine against Tb is *Mycobacterium bovis* Bacillus Calmette-Guérin (BCG); however, it is only partially effective: it provides protection against severe forms of Tb in infants but is unable to prevent the development of adult pulmonary Tb, the most prevalent form of the disease (2, 3). Thus, there is an urgent need to develop novel vaccine strategies that are safe and effective and can prevent all forms of Tb in different age groups.

Protection against Tb has long been attributed to CD4^+^ T cells and in particular to IFN-γ-secreting T-helper 1 (Th1) cells (4). However, recent knowledge suggests that additional pathways could also play important roles in vaccine-induced immunity against Tb. In this respect, IL-23-driven Th17 cells were shown to contribute to the generation of antigen (Ag)-specific Th1 cells and the protection against *Mycobacterium tuberculosis* (*Mtb*) following vaccination with BCG (5) and proved to be key effector cells in different parenteral and mucosal subunit-based Tb vaccine models (6–9). Furthermore, BCG- or environmental mycobacteria-induced regulatory T cells (Tregs) have been proposed as one of the reasons for the delayed onset of adaptive immunity observed in Tb and to limit the generation of sterilizing immunity (10, 11).

Dendritic cells (DCs) are specialized Ag-presenting cells that play a central role in initiating and regulating adaptive immunity (12). Owing to their potent Ag presentation capacity and ability to generate distinct T-cell responses, efficient and specific delivery of Ags to DCs is the cornerstone for generating Ag-specific effector and memory cells against tumors or pathogens (13, 14). Administration of autologous DCs exogenously loaded with tumor-Ags was the first DC-based vaccine developed (15). Since then, a few other *ex vivo* DC vaccines have been generated and tested in clinical trials. However, they show low clinical responses and have high production costs, making them unavailable for mass vaccination in developing countries which hold the highest Tb burden (16, 17). To overcome these limitations, a new concept of directly targeting endocytic receptors on DCs by Ag-coupled antibodies or glycosylated molecules was developed as a more effective strategy. Moreover, this type of approach allows the targeting of specific DC subsets while maintaining the natural environment of the cells (13, 17, 18).

C-type lectin receptors (CLRs) are an important family of calcium-dependent lectins that are structurally related through the expression of at least one carbohydrate recognition domain (CRD). Many CLRs are abundantly but also uniquely expressed on the surface of specific DC subsets, where they mediate pathogen recognition and internalization of Ags (19, 20). Due to these properties, CLRs represent ideal candidates for targeting purposes. Pioneer studies in this field focused on the use of antibodies against DEC-205 (CD205) conjugated to OVA to elicit resistance against OVA-modified pathogens and tumors (21–23). However, expression of DEC-205 in humans is not only restricted to DCs (24), thus carrying the possibility of inadvertently targeting other cell types. In contrast, human DC-specific-ICAM3-grabbing-nonintegrin (DC-SIGN, CD209) is predominantly present on the surface of immature monocyte-derived DCs and at lower levels on mature monocyte-derived DCs and macrophages in the skin, mucosal tissues, and secondary lymphoid organs (25, 26). Contrary to humans, who only express DC-SIGN, mice possess eight DC-SIGN homologs in their genome. Sequence analysis of the DC-SIGN receptor family in humans and mice has demonstrated that it underwent substantial divergence between both species. Thus, none of the murine DC-SIGN homologs presents the same functions (glycan specificity, internalization and intracellular trafficking, intercellular adhesion and signaling) as the human DC-SIGN, making the study of this receptor in mice challenging (27, 28). To circumvent this issue, we generated and made use of the hSIGN mouse model which expresses human DC-SIGN under the control of the murine CD11c promoter and thus expresses the human receptor predominantly on DCs (29). We previously demonstrated that DC targeting *via* injection of anti-DC-SIGN antibodies into hSIGN mice induces strong and durable Ag-specific CD4^+^ and CD8^+^ T-cell responses capable of mediating protection against infection with OVA-expressing *Listeria monocytogenes* (30). Thus, this study provided powerful evidence that targeting of DC-SIGN *in vivo* results in protection against intracellular pathogens.

Targeting of DCs *via* anti-CLR antibodies is also known to induce tolerance unless an adjuvant is co-delivered (21, 31, 32). Given that adjuvants have the ability of skewing the type of response upon vaccination by the induction of different T-helper subsets, selection of the proper adjuvant system is critical for targeting approaches. In the current study, we aimed to develop a new vaccine strategy against *Mtb* based on targeting DCs through the use of anti-human-DC-SIGN antibodies conjugated to Ag85B, a subdominant but highly immunogenic protein from *Mtb* (33), and peptide 25 (P25) (covering the amino-acid residues 240–254) of Ag85B, a major Th1 epitope (34). We provide here the proof of concept that immunization with anti-DC-SIGN antibodies conjugated to *Mtb* Ags can effectively induce anti-mycobacterial immunity *in vivo*. Furthermore, we characterize the type of response elicited by different adjuvant systems.

## Materials and Methods

### Mice

hSIGN mice were described previously (29) and P25ktk mice (35) were obtained from Jackson Laboratories. P25ktk mice were further crossed to CD45.1 mice. Sex- and age-matched mice between 12 and 18 weeks were used in all experiments. All animals were bred and maintained under specific pathogen-free conditions at the animal facility of TWINCORE, Center for Experimental and Clinical Infection Research (Hannover, Germany) or the Helmholtz Center for Infection Research (HZI, Braunschweig, Germany). All animal experiments were approved by the Veterinary Institute of LAVES (Lower Saxony State Office for Consumer Protection and Food Safety, permit numbers: 12/0732 and 17/2472) considering the German Animal Welfare Act.

### Conjugation of anti-DC-SIGN Antibodies

The conjugated monoclonal anti-DC-SIGN (αDC-SIGN) antibodies (clone: AZN-D1, IgG1) to Ag85B protein (αDC-SIGN:Ag85B) and Ag85B_240–254_ peptide (P25) (αDC-SIGN:P25) were prepared as previously described (25). Briefly, the antibodies or an isotype control antibody were conjugated to the different proteins using the crosslinking agent sulfosuccinimidyl-4-(N-maleimidomethyl)-cyclohexane-1-carboxylate according to the manufacturer’s protocol (sulfo-SMCC; Pierce).

### Flow Cytometry

The following antibodies and reagents were purchased from Thermo Fisher Scientific/eBioscience: anti-CD4 (GK1.5), anti-CD4 (RM4–5), anti-CD45.1 (A20), anti-IFN-γ (XMG1.2), anti-CD11c (N418), anti-MHC-II (M5/114.15.2), anti-CD86 (GL1), anti-IL-17A (eBio17B7), anti-CD44 (IM7), anti-IL-2 (JESG-SH4), anti-TNF-α (MP6-XT22), anti-IL-10 (JES5-16E3), anti-FoxP3 (FJK-16s), anti-KLRG1 (2F1), anti-CD127 (A7R34), and Brefeldin A. Cellular aggregates were excluded by gating singlets using SSC-A versus SSC-W. Dead cells were excluded by LIVE/DEAD^®^ Fixable Aqua Dead (Thermo Fisher Scientific/Invitrogen) cell staining. For intracellular cytokine staining, cells were fixed with 0.5% Paraformaldehyde (Roth) overnight and permeabilized in PBA-S buffer (0.5% Saponin (Roth) and 0.25% BSA (Roth) in PBS). Intranuclear FoxP3 staining was performed using the Fixation/Permeabilization kit (Thermo Fisher Scientific/eBioscience) according to manufacturer’s instructions. Data acquisition was performed using a LSRII (BD, Biosciences) or a CyAn™ ADP (Beckman Coulter) flow cytometer. Data analysis was performed with FlowJo software (Tree Star).

### Adjuvants

Zymosan was purchased from Sigma-Aldrich and prepared as indicated by the manufacturer’s instructions. The non-toxic CTA1-DD adjuvant was kindly provided by Prof. N. Lycke (Department of Clinical Immunology, Göteborg University, Sweden).

### *In Vitro* T-Cell Proliferation Assay

Granulocyte-macrophage colony-stimulating factor (GM-CSF)-derived bone-marrow-derived dendritic cells (BMDCs) were generated from BM cells using a standard protocol. Briefly, BM cells were cultured for 7 days in complete RPMI [10% FCS (Biochrom), 10 mM Hepes (Gibco), 50 µM β-mercaptoethanol (Gibco), 100 U/mL penicillin, and 100 μg/mL streptomycin (Biochrom)] supplemented with 5% culture supernatant of a GM-CSF-producing cell line (36). On day 7, 25.000 BMDC/well were incubated with αDC-SIGN:Ag85B or αDC-SIGN:P25 at the indicated concentrations in the presence of αCD40 (1 µg/mL; clone 1C10) for 24 h, washed and incubated in a 1:8 ratio with CD4^+^ T cells obtained from the spleen and lymph nodes of P25ktk mice, enriched by negative magnetic selection using the Dynabeads™ Untouched™ Mouse CD4 Cells isolation kit (Thermo Fisher Scientific/Invitrogen) following the manufacturer’s instructions. After enrichment, cells were labeled with the CellTrace Violet Cell Proliferation Kit (Thermo Fisher Scientific/Invitrogen). The purity of enrichment was checked by flow cytometry and resulted higher than 85%. Co-cultures were then incubated in complete RPMI medium for 4 days at 37°C in 96-well round bottom plates (Greiner Bio-One/Cellstar). At day 4, cells were stimulated with PMA (0.1 µg/mL) and ionomycin (1 µg/mL). After 2 h of stimulation, Brefeldin A was added for additional 2 h before staining for flow cytometric analysis.

### *In Vitro* Stimulation of BMDCs

GM-CSF-derived BMDCs were generated as mentioned above and stimulated with αCD40 (1 µg/mL) or different concentrations of CTA1-DD or zymosan for 24 h followed by staining for surface activation markers for flow cytometric analysis. LPS (100 ng/mL; *E. coli* Serotype 055:B5; Merck/Sigma Aldrich) was used as positive control. Culture supernatants were collected and ELISA assays were performed to determine IL-6, IL-23, IL-1β, and IL-10 production following the manufacturer’s instructions (R&D System).

### *In Vivo* T-Cell Priming

2 × 10^6^ CellViolet-labeled congenic CD45.1^+^ CD4^+^ P25ktk T cells were adoptively transferred intravenously (i.v.) into WT or hSIGN mice. One day later, mice were immunized with αDC-SIGN:P25 (2 μg/mouse), αDC-SIGN:Ag85B (2 μg/mouse) or isotype control (2 μg/mouse) in the presence of αCD40 (10 μg/mouse), CTA1-DD (10 μg/mouse) or zymosan (200 μg/mouse). Five days after transfer, spleens were removed and stimulated with PMA (0.1 µg/mL) and ionomycin (1 µg/mL). After 2 h of stimulation, Brefeldin A was added for additional 2 h before staining for flow cytometric analysis.

### Vaccination with αDC-SIGN Antibodies

WT or hSIGN mice were immunized with αDC-SIGN:P25 (10 μg/mouse) in combination with αCD40 (10 μg/mouse), CTA1-DD (10 μg/mouse) or zymosan (200 μg/mouse) intraperitoneally (i.p.) three times with a 2-week interval between each immunization. Unvaccinated controls received saline solution (PBS).

### Experimental Infections

Vaccinated mice were challenged 42 days after the first immunization by i.v. administration of 2 × 10^6^ colony-forming units (CFUs) of *M. bovis* BCG overexpressing Ag85B (*M. bovis* BCG-Ag85B), kindly provided by Dr. Joel Ernst (NYU School of Medicine, USA). *M. bovis* BCG-Ag85B was grown at 37°C in Middlebrook 7H9 broth (BD Bisociences) supplemented with 10% Middlebrook oleic acid–albumin–dextrose–catalase (OADC) enrichment medium (Difco Laboratories), 0.05% of Tween 80 (Roth) and 0.002% glycerol (Roth). After 5 days of infection, mice were sacrificed and spleens were collected in sterile bags (Nasco) containing 1 mL of WTA buffer [0.01% Tween-80 and 0.05% BSA (Roth)] and mechanically disrupted. Viable bacterial loads were determined by plating serial dilutions onto Middlebrook 7H11 agar (BD Biosciences) supplemented with 10% OADC (Difco Laboratories) and 0.5% glycerol (Roth). Colonies were counted after 2 to 3 weeks of incubation at 37°C.

### Statistical Analysis

Data analysis was performed using GraphPad Prism Software 5.0. Statistics were calculated using one-way or two-way ANOVA as indicated in figure legends. *P-*Values were considered significant as follows: *p < 0.05, **p < 0.01, and ***p < 0.001.

## Results

### Targeting *Mtb* Ags to DCs *via* DC-SIGN Induces Strong CD4^+^ T-Cell Responses

**Figure 1:**
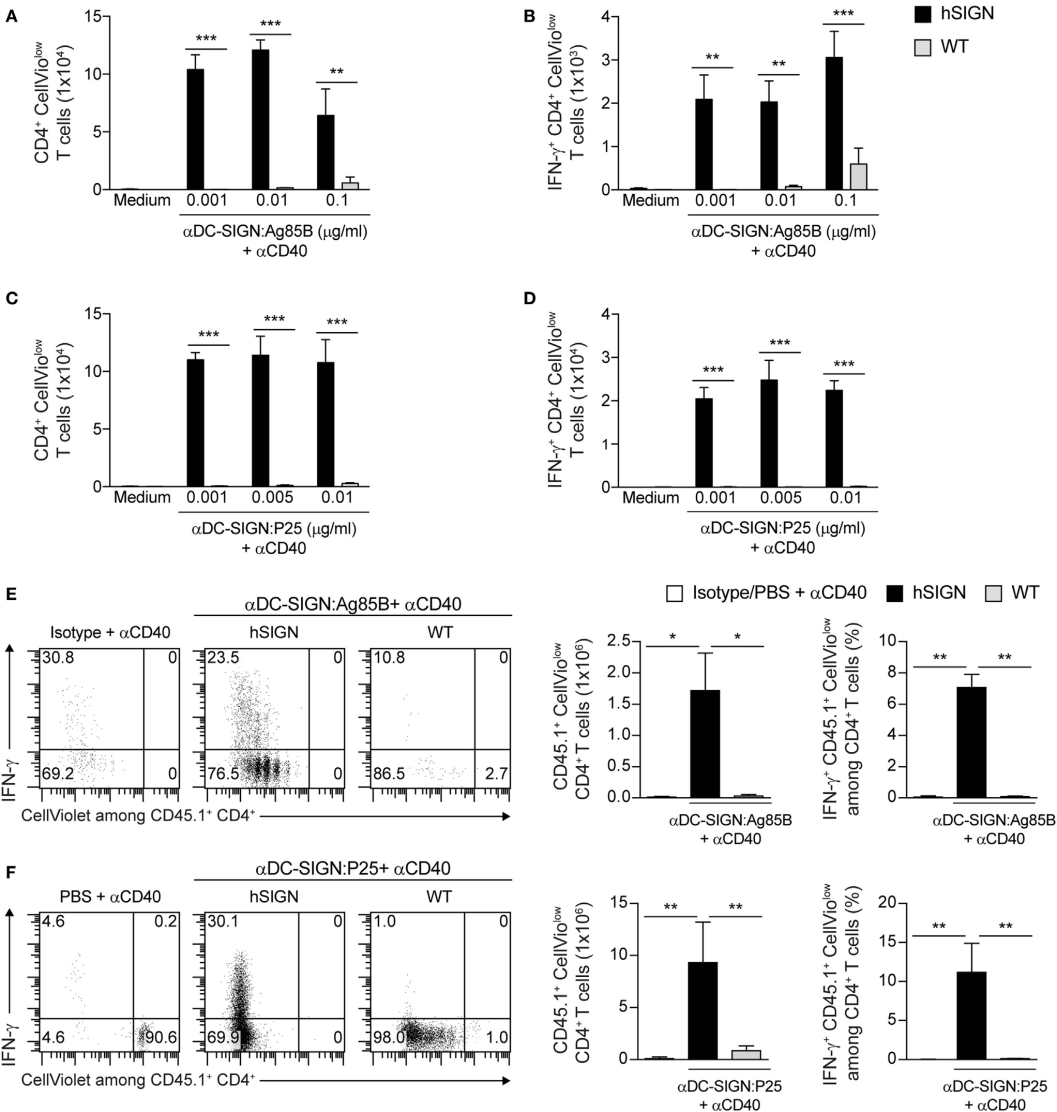
*In vitro* and *in vivo* targeting of *Mycobacterium tuberculosis* antigens to DC *via* DC-specific-ICAM3-grabbing-nonintegrin (DC-SIGN) induces strong CD4^+^ T-cell responses. **(A–D)** CellViolet-labeled CD4^+^ P25ktk T cells were co-cultured with WT or hSIGN BMDC targeted with increasing doses of **(A,B)** αDC-SIGN:Ag85B or **(C,D)** αDC-SIGN:P25 in the presence of αCD40 (1 µg/mL). After 4 days of co-culture **(A,C)** *in vitro* proliferation of CD4^+^ T cells and **(B,D)** intracellular IFN-γ production were determined by flow cytometry after restimulation with PMA/ionomycin. Bar graphs represent the **(A,C)** number of proliferating CellVio^low^ CD4^+^ cells and **(B,D)** number of proliferating IFN-γ^+^ CellVio^low^ CD4^+^ cells. Error bars represent SD of triplicate wells from one of three experiments. **p < 0.01 and ***p < 0.001; two-way ANOVA with Bonferroni’s *post hoc* test. **(E,F)** CellViolet-labeled CD45.1^+^ CD4^+^ P25ktk T cells were adoptively transferred into WT or hSIGN mice. One day later, mice were immunized with **(E)** αDC-SIGN:Ag85B (2 µg), **(F)** αDC-SIGN:P25 (2 µg) or an isotype control (2 µg) **(E)** or vehicle **(F)** in the presence of αCD40 (10 µg). Four days post-immunization **(E,F)** *in vivo* cell proliferation and intracellular IFN-γ production were determined by flow cytometry in splenocytes after *ex vivo* restimulation with PMA/ionomycin. Shown are representative flow cytometry plots depicting the percentage of IFN-γ^+^ CellVio^low^ among CD45.1^+^ CD4^+^ T cells. Bar graphs represent the total number of proliferating CD45.1^+^ CellVio^low^ CD4^+^ cells and percent of proliferating IFN-γ^+^ CD45.1^+^ CellVio^low^ among all live CD4^+^ T cells. Error bars represent SD of 3–5 mice per group from one of two experiments. *p < 0.05, **p < 0.01, and ***p < 0.001; one-way ANOVA with Bonferroni’s *post hoc* test.

We previously showed that αDC-SIGN antibodies conjugated to OVA induce strong and persistent Ag-specific CD4^+^ and CD8^+^ T-cell responses which promote rapid clearance of OVA-expressing *Listeria monocytogenes* infection (30). Thus, we proposed DC targeting *via* DC-SIGN as a promising strategy for vaccination protocols against intracellular pathogens. In order to determine whether this strategy could be effective against *Mtb*, we conjugated αDC-SIGN antibodies to Ag85B (αDC-SIGN:Ag85B) and Ag85B_240–254_ peptide (P25) (αDC-SIGN:P25) and tested their capacity to induce T-cell activation *in vitro*. To achieve this, BMDCs were prepared from WT or hSIGN mice and pulsed with different concentrations of αDC-SIGN:Ag85B or αDC-SIGN:P25 in the presence of αCD40 as an adjuvant. The capacity of antibody-targeted WT and hSIGN BMDCs to promote T-cell responses was then evaluated by co-culturing them with CellViolet-labeled CD4^+^ T cells purified from P25ktk mice, carrying a transgenic TCR that specifically reacts to P25 in the context of MHC class-II presentation (35). After 4 days of co-culture, cell proliferation and cytokine production were measured by flow cytometry as parameters of T-cell activation. Both αDC-SIGN:Ag85B and αDC-SIGN:P25 in combination with αCD40 were able to promote Ag presentation by BMDCs generated from hSIGN but not WT mice, as evidenced by their ability to induce the proliferation of CD4^+^ P25ktk T cells (Figures 1A,C). Moreover, both conjugated antibodies also promoted the production of IFN-γ in the proliferating CD4^+^ P25ktk T cells, indicating that targeting DCs *via* DC-SIGN not only results in Ag presentation to CD4^+^ T cells *in vitro* but also in efficient cytokine production (Figures 1B,D).

We next determined whether targeting Ags to DCs *via* human DC-SIGN also leads to enhanced T-cell responses *in vivo*. To this aim, we adoptively transferred CellViolet-labeled congenic CD45.1^+^ CD4^+^ P25ktk T cells into WT or hSIGN-recipient mice and 1 day later immunized them with either αDC-SIGN:Ag85B, αDC-SIGN:P25, isotype or vehicle control in combination with αCD40. Four days after immunization, mice were sacrificed and the proliferation as well as cytokine production of the transferred T cells was determined by flow cytometry after gating on CD45.1^+^ CD4^+^ T cells in the splenic cell population. Treatment of hSIGN mice with either αDC-SIGN:Ag85B or αDC-SIGN:P25 led to significant expansion and IFN-γ production of the transferred CD45.1^+^ CD4^+^ P25ktk T cells. In contrast, in WT mice only a marginal expansion of Ag-specific CD4^+^ T cells was observed upon immunization, indicating that the conjugated Ags were preferentially delivered to DCs *via* the DC-SIGN receptor. Similar results were obtained after the administration of an isotype antibody in combination with αCD40 while the vehicle control failed to induce T-cell proliferation (Figures 1E,F). Thus, *in vitro* and *in vivo* targeting of DCs through αDC-SIGN antibodies conjugated to *Mtb* Ags efficiently promotes Ag presentation, proliferation and IFN-γ production by CD4^+^ T cells.

### CTA1-DD and Zymosan Induce DC Activation and Cytokine Production

**Figure 2:**
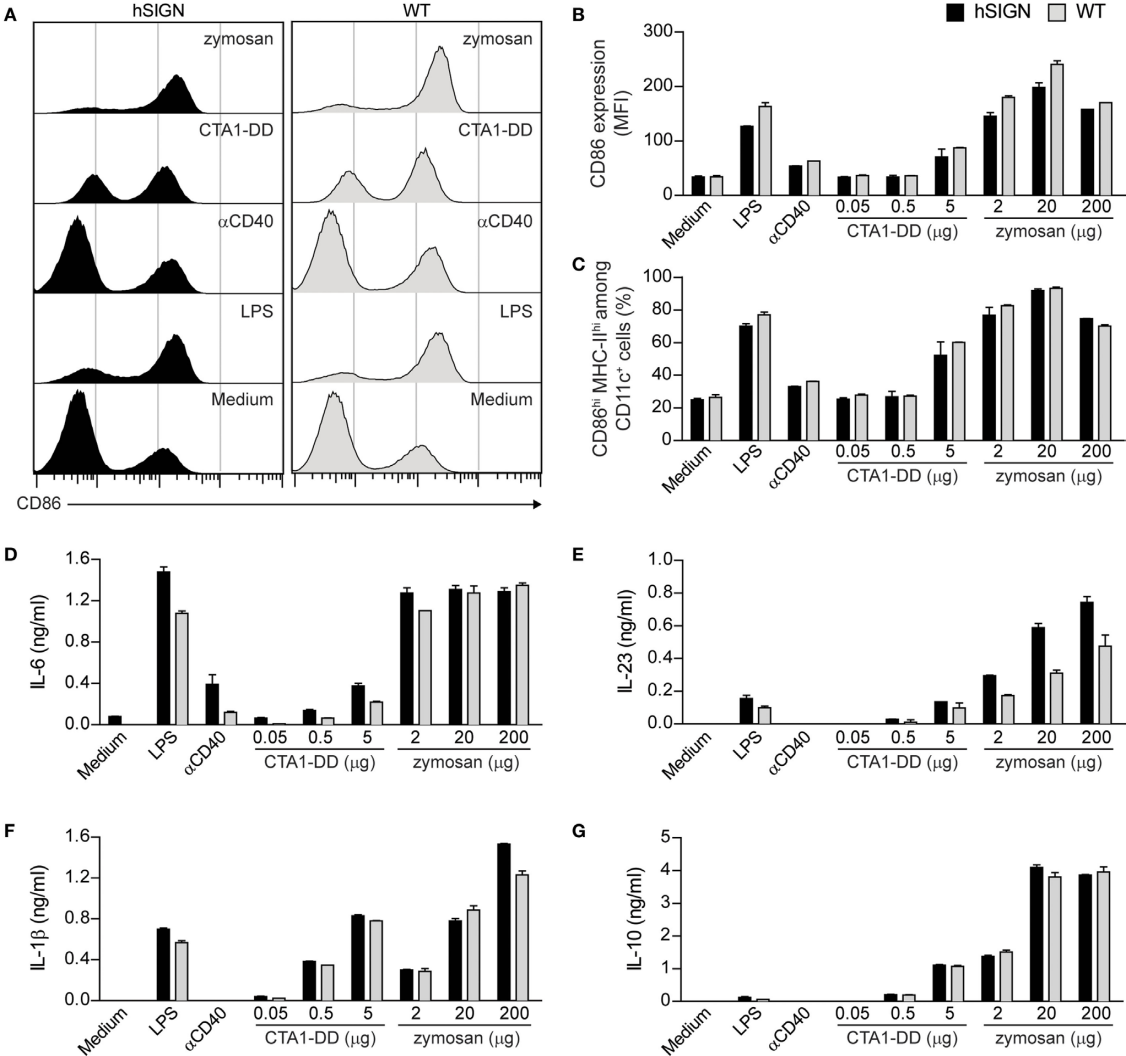
CTA1-DD and zymosan induce DC activation and cytokine production. **(A–G)** WT or hSIGN BMDC were treated with αCD40 (1 µg/mL) and different concentrations of CTA1-DD and zymosan. LPS (100 ng/mL) was used as a positive control. After 24 h stimulation, **(A–C)** CD86 and MHC-II surface expression was determined by flow cytometry and **(D)** IL-6, **(E)** IL-23, **(F)** IL-1β and **(G)** IL-10 production by ELISA. **(A)** Representative histograms from one of three independent experiments. **(B)** Bar graphs represent mean fluorescence intensity (MFI) of CD86 and **(C)** percent of CD86^hi^ MHC-II^hi^ among CD11c^+^ cells. Error bars represent SD of triplicate wells from one of three experiments.

In recent years, it has been demonstrated that vaccine-induced immunity against *Mtb* not only depends on the generation of Th1 cells but also on other T-helper subsets such as Th17 cells (5, 7). Thus, adjuvant selection for new vaccine candidates is critical. Cholera toxin (CT) is a potent cyclic adenosine monophosphate (cAMP)-based adjuvant which can effectively prime Th17 cells *via* stimulation of CD11b^+^ DCs (37). Unfor-tunately, CT is not approved for human vaccination due to its high toxicity. To solve this problem, a fusion protein between the A1 catalytic domain of CT and two immunoglobulin-binding D regions from *Staphylococcus aureus* protein A called CTA1-DD was developed with the same adjuvant activity of CT but without its toxicity (38). Through a different mechanism, the *Saccharomyces cerevisiae* cell-wall component zymosan also promotes strong Th17 cell responses using TLR-dependent and -independent pathways (39–41). Therefore, we evaluated the ability of these adjuvants to broaden the spectrum of T-helper responses elicited by our αDC-SIGN antibodies. We first tested whether CTA1-DD and zymosan were able to induce DC maturation and cytokine production. For this, we generated BMDCs from WT and hSIGN mice and incubated them with αCD40 (1 µg/mL), LPS (100 ng/mL) and different concentrations of CTA1-DD and zymosan. After 24 h, upregulation of the activation marker CD86 was measured by flow cytometry and cytokine production by ELISA. CTA1-DD only mildly enhanced CD86 surface expression by WT and hSIGN DCs (Figures 2A,B) but was able to increase the percentage of CD86^hi^ MHC-II^hi^ DCs of both genotypes at the highest concentration tested (Figure 2C). Moreover, CTA1-DD also promoted the secretion of IL-6, IL-23 and IL-1β in a dose-dependent manner while inducing low levels of IL-10 (Figures 2D–G). In contrast, zymosan significantly upregulated CD86 expression (Figures 2A,B) and increased the percentage of CD86^hi^ MHC-II^hi^ DCs of both genotypes at all concentrations tested (Figure 2C). Overall, zymosan induced the highest cytokine levels (Figures 2D–G). Surprisingly, αCD40 proved a poor stimulus for DC activation, at least at the studied concentration (Figures 2A–G). As expected, the TLR4 agonist LPS served as positive control promoting DC maturation and cytokine production (Figures 2A–G). In addition, no marked differences were observed between WT and hSIGN BMDCs in regard to their ability to upregulate CD86 expression and secrete pro- and anti-inflammatory cytokines, demonstrating that human DC-SIGN expression does not affect DC function/activation. Taken together, these results indicate that both CTA1-DD and zymosan are capable of promoting DC activation and cytokine production.

### Administration of αDC-SIGN:P25 with CTA1-DD or Zymosan Promotes Effective T-Cell Priming

**Figure 3:**
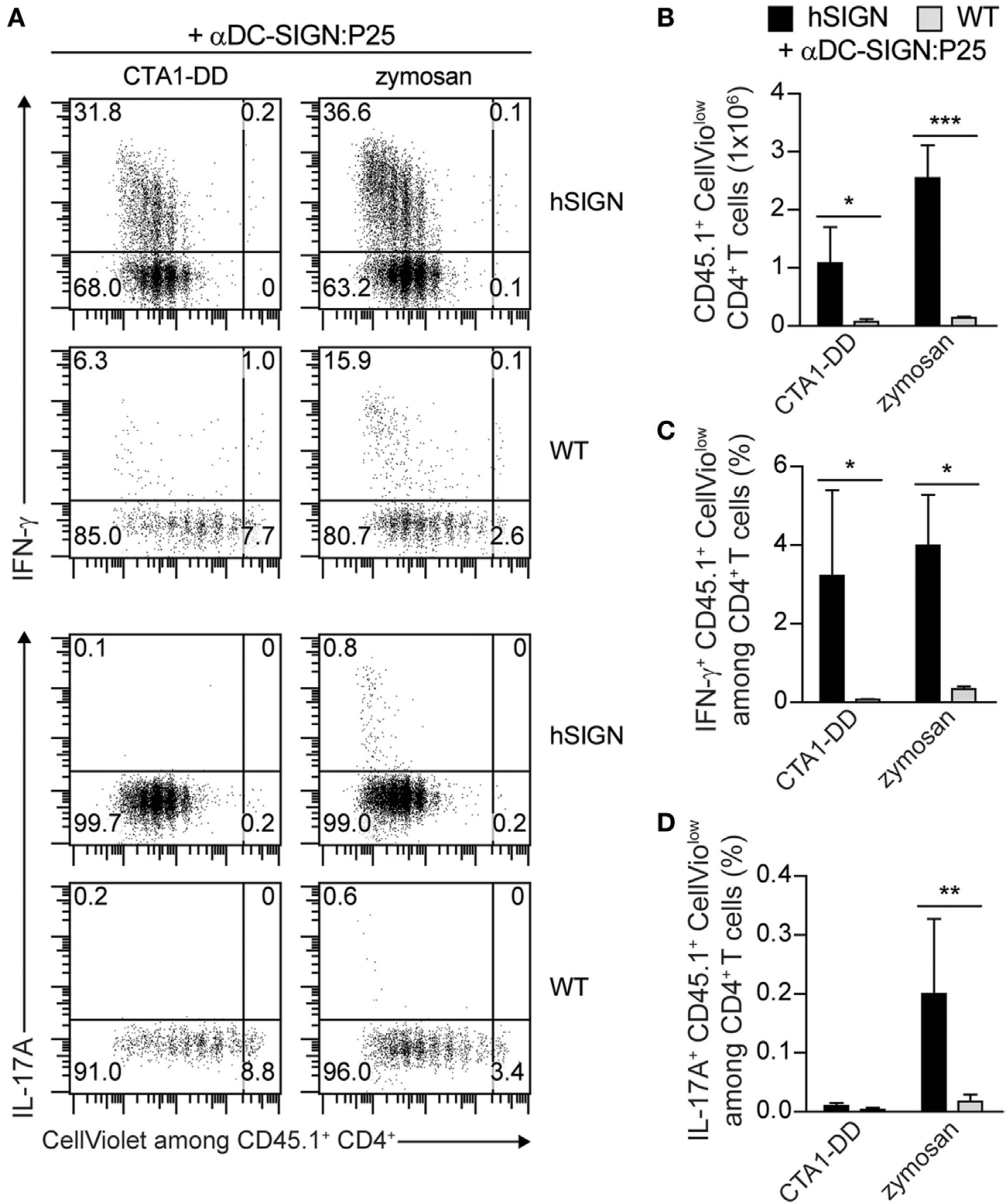
Administration of αDC-SIGN:P25 with CTA1-DD or zymosan promotes T-cell priming. **(A–D)** CellViolet-labeled CD45.1^+^ CD4^+^ P25ktk T cells were adoptively transferred into WT or hSIGN mice. One day later, mice were immunized with αDC-SIGN:P25 (2 µg) plus CTA1-DD (10 µg) or zymosan (200 µg). Four days post-immunization **(A,B)** *in vivo* cell proliferation and intracellular **(A,C)** IFN-γ and **(A,D)** IL-17A production were determined by flow cytometry in splenocytes after *ex vivo* restimulation with PMA/ionomycin. **(A)** Representative flow cytometry plots depicting the proliferation and percentage of IFN-γ^+^ (upper panels) or IL-17A^+^ (lower panels) CellVio^low^ among CD45.1^+^ CD4^+^ T cells. Bar graphs represent the **(B)** total number of proliferating CD45.1^+^ CellVio^low^ CD4^+^ cells and percent of proliferating **(C)** IFN-γ^+^ or **(D)** IL-17A^+^ CD45.1^+^ CellVio^low^ among all live CD4^+^ T cells. Error bars represent SD of three mice per group from one of two experiments. *p < 0.05, **p < 0.01, and ***p < 0.001; two-way ANOVA with Bonferroni’s *post hoc* test.

We next determined whether CTA1-DD and zymosan in combination with the αDC:SIGN conjugates could also induce specific T-cell responses *in vivo*. Given the similar performance observed in our preliminary results, we opted to focus on the αDC-SIGN:P25 antibody for further experiments. To investigate this, we performed adoptive transfer assays as described above using CellViolet-stained congenic CD45.1^+^ CD4^+^ P25ktk T cells transferred into WT or hSIGN-recipient mice. One day later, we injected αDC-SIGN:P25 in the presence of CTA1-DD or zymosan. Expansion and cytokine production by the transferred cells was evaluated 4 days later by flow cytometry. Immunization of hSIGN but not WT mice with αDC-SIGN:P25 plus either CTA1-DD or zymosan significantly induced expansion and IFN-γ production of the transferred CD45.1^+^ CD4^+^ P25ktk T cells (Figures 3A–C). However, only zymosan was able to significantly enhance IL-17A production by the transferred CD4^+^ T cells (Figure 3D). Thus, targeting DC-SIGN using a P25-conjugated αDC-SIGN antibody co-delivered with CTA1-DD or zymosan promotes the generation and proliferation of Ag-specific Th1 cells. Furthermore, zymosan can, in addition, slightly prime Th17 cells.

### Vaccination with αDC-SIGN:P25 Plus αCD40, CTA1-DD, or Zymosan Does Not Induce Th17 Cells but Prevents the Expansion of Tregs

**Figure 4:**
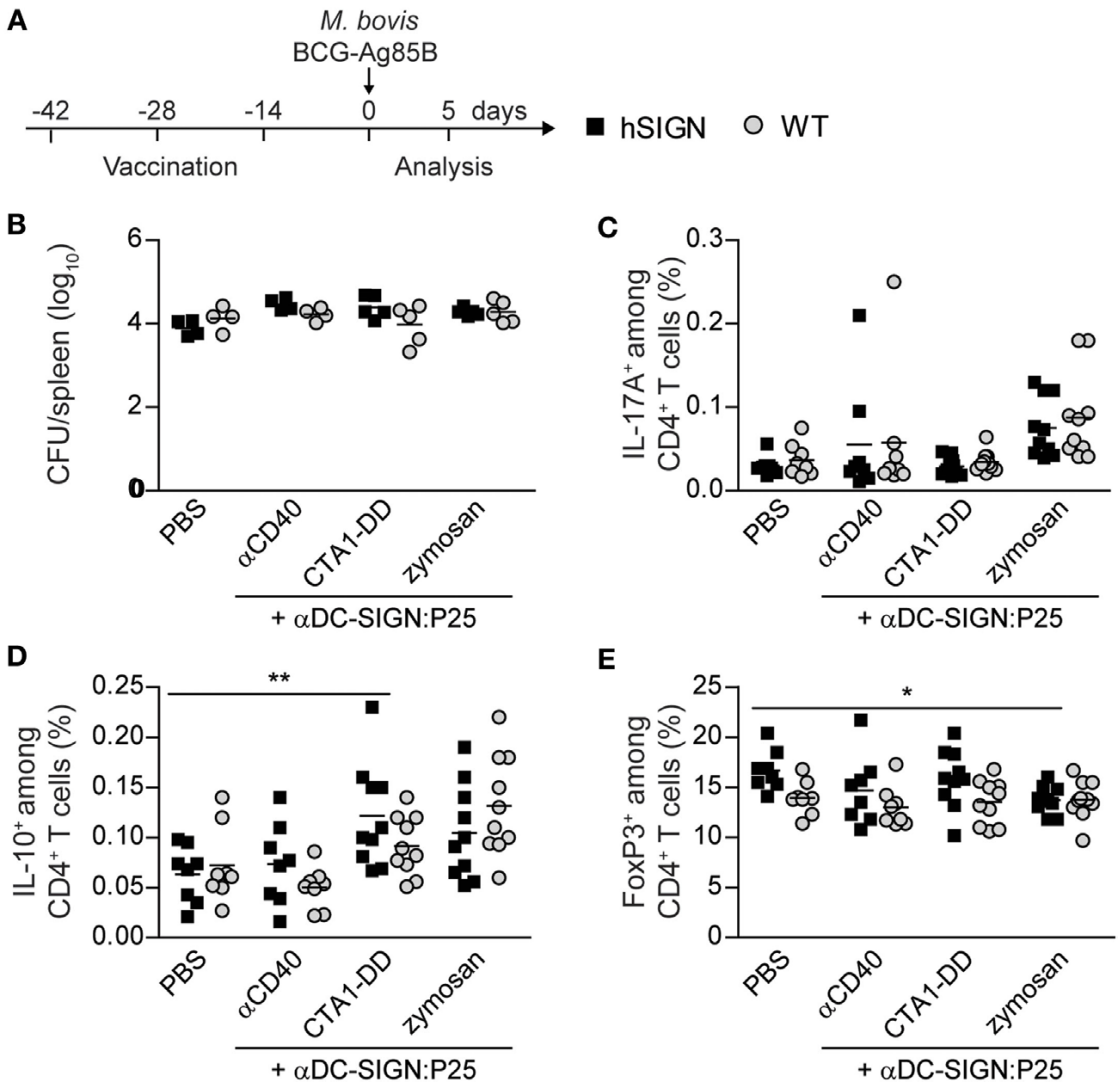
Vaccination with αDC-SIGN:P25 plus αCD40, CTA1-DD or zymosan does not induce Th17 cells but prevents the expansion of regulatory T cells. **(A)** Experimental scheme. WT or hSIGN mice were vaccinated i.p. at days −42, −28, and −14 with vehicle (PBS) or αDC-SIGN:P25 (10 µg) plus αCD40 (10 µg), CTA1-DD (10 µg) or zymosan (200 µg). At day 0, mice were challenged i.v. with 2 × 10^6^ *Mycobacterium bovis* BCG-Ag85B. Five days later, bacterial burden, intracellular cytokine production after *ex vivo* restimulation with P25 (30 µg/mL), and FoxP3 expression were determined in spleen. **(B)** Graph represents the logarithmic transformation of the number of colony-forming units (CFUs) per spleen of vaccinated mice. **(C–E)** Graphs represent percent of **(C)** P25-specific IL-17A^+^, **(D)** IL-10^+^ and **(E)** FoxP3^+^ among CD4^+^ T cells of vaccinated mice. Each symbol represents an individual mouse and results are pooled from two experiments with 4–5 mice per group. *p < 0.05 and **p < 0.01; two-way ANOVA with Bonferroni’s *post hoc* test.

Having demonstrated the potential of αDC:SIGN antibodies to target DC and induce strong Ag-specific T-cell responses, we decided to evaluate the αDC-SIGN:P25 antibody in a standard vaccination protocol. For this, we immunized WT or hSIGN mice i.p. at days −42, −28, and −14 prior to *M. bovis* BCG-Ag85B infection with αDC-SIGN:P25 (10 µg) plus either αCD40 (10 µg), CTA1-DD (10 µg) or zymosan (200 µg). As control group, mice were treated with vehicle (PBS). At day 0, mice were infected i.v. with 2 × 10^6^ *M. bovis* BCG-Ag85B, a strain that overexpresses Ag85B. Five days later, bacterial loads and intracellular cytokine production after *ex vivo* restimulation with the cognate peptide P25 were determined in spleens (Figure 4A). Contrary to our short-term vaccination experiments, we could not detect any differences in bacterial burden nor a significant increase in the percentage of P25-specific IL-17A-producing CD4^+^ T cells in hSIGN mice vaccinated with the αDC-SIGN:P25 antibody plus any of the adjuvants used (Figures 4B,C). These results seem to indicate that in the tested settings the αDC-SIGN:P25 antibody is unable to promote a persistent Th17 response and does not impact bacterial growth. On the other hand, limited vaccine efficacy is thought to be linked to the expansion of Tregs upon BCG administration (10). Therefore, vaccine candidates should avoid induction of anti-inflammatory immune responses. Thus, we also evaluated whether vaccination with αDC-SIGN:P25 plus αCD40, CTA1-DD or zymosan could increase the population of IL-10-producing T cells or FoxP3^+^ Tregs in spleens. hSIGN mice vaccinated with CTA1-DD showed a significant increase in the percentage of P25-specific IL-10^+^ CD4^+^ T cells only compared with unvaccinated controls (Figure 4D). Yet, we could not detect differences in the percentage of IL-10-producing CD4^+^ T cells in any of the other experimental groups (Figure 4D). Regarding Tregs, hSIGN mice vaccinated in the presence of zymosan showed a significant decrease in FoxP3^+^ CD4^+^ T cells compared with PBS-treated controls (Figure 4E). None of the other adjuvant systems employed showed differences in this population compared with controls (Figure 4E), suggesting that Tregs are not significantly expanded during our vaccina-tion approach.

### Vaccination With αDC-SIGN:P25 Plus αCD40, CTA1-DD, or Zymosan Induces Pro-inflammatory Cytokine Production

**Figure 5:**
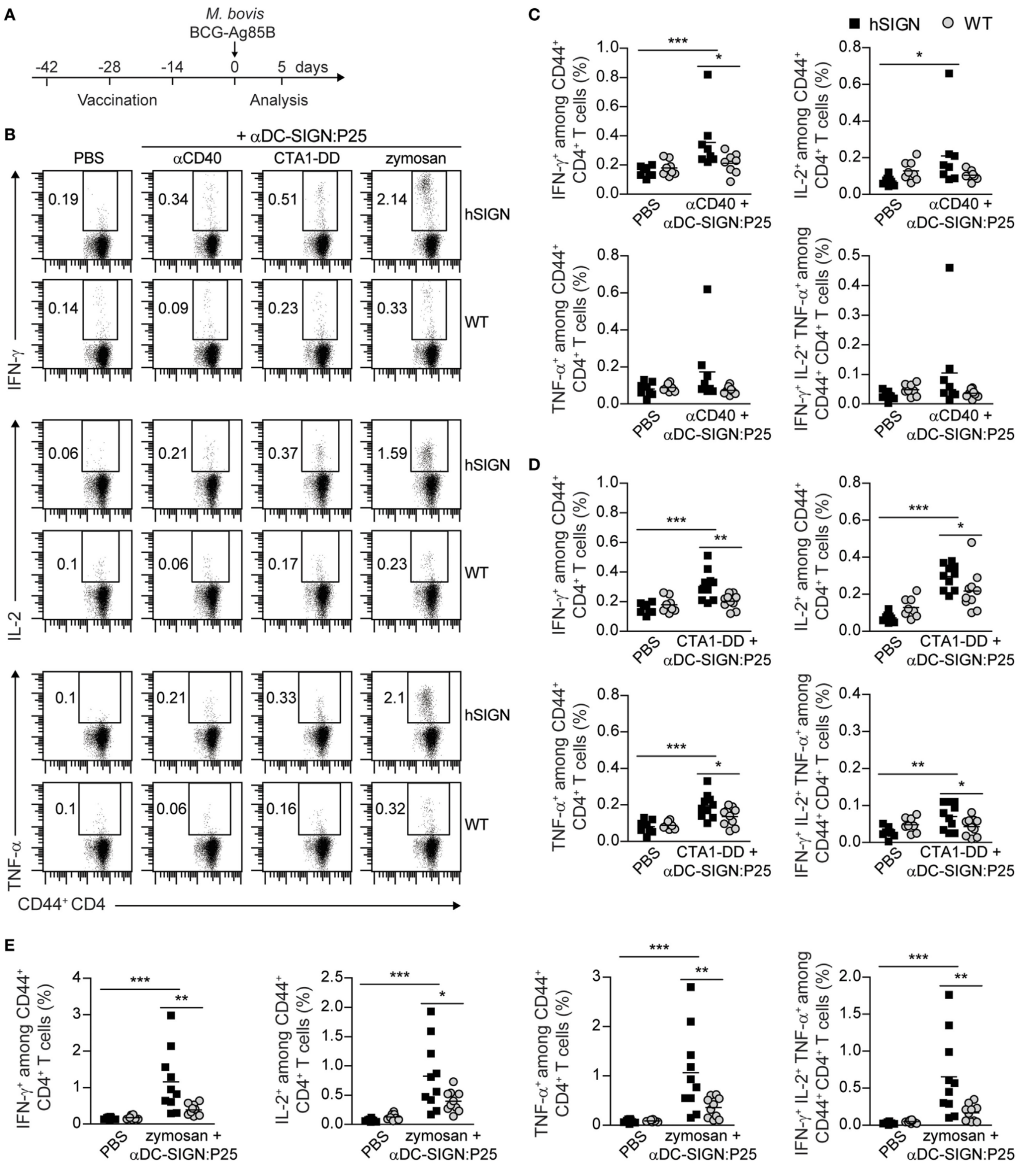
Vaccination with αDC-SIGN:P25 plus αCD40, CTA1-DD or zymosan induces pro-inflammatory cytokine production. **(A)** Experimental scheme. WT or hSIGN mice were vaccinated i.p. at days −42, −28, and −14 with vehicle (PBS) or αDC-SIGN:P25 (10 µg) plus αCD40 (10 µg), CTA1-DD (10 µg) or zymosan (200 µg). At day 0, mice were challenged i.v. with 2 × 10^6^ *Mycobacterium bovis* BCG-Ag85B. Five days later, mice were sacrificed and splenocytes were isolated and stained for intracellular cytokine production after *ex vivo* restimulation with P25 (30 µg/mL). **(B)** Representative flow cytometry plots depicting the percentage of IFN-γ^+^ (upper panels), IL-2^+^ (middle panels) and TNF-α^+^ (lower panels) among CD44^+^ CD4^+^ T cells. **(C–E)** Graphs represent percent of IFN-γ^+^, IL-2^+^, TNF-α^+^ and polyfunctional (IFN-γ^+^ IL-2^+^ TNF-α^+^) among CD44^+^ CD4^+^ T cells of mice vaccinated with **(C)** αCD40, **(D)** CTA1-DD and **(E)** zymosan. Each symbol represents an individual mouse and results are pooled from two experiments with 4–5 mice per group. *p < 0.05, **p < 0.01, and ***p < 0.001; two-way ANOVA with Bonferroni’s *post hoc* test.

T-helper 1 cells have a preponderant role in mounting protective immune responses against Tb (42, 43). Polyfunctional T cells, defined as able to produce multiple pro-inflammatory cytokines, such as IFN-γ, IL-2 and TNF-α, have been identified as correlates of protection in the mouse model of Tb (44, 45). We therefore tested the ability of the αDC-SIGN:P25 antibody in combination with αCD40, CTA1-DD or zymosan to induce this type of responses after vaccination and *ex vivo* restimulation with P25 (Figure 5A). hSIGN mice vaccinated with αDC-SIGN:P25 plus αCD40 only showed a mild increase in the percentage of Ag-specific IFN-γ and IL-2-producing cells among CD44^+^ CD4^+^ T cells (Figures 5B,C). In the case of CTA1-DD, we could observe a significant increase in the percentage of IFN-γ, IL-2 and TNF-α-producing cells among CD44^+^ CD4^+^ T cells in hSIGN mice compared with WT and unvaccinated controls (Figures 5B,D). Moreover, we could observe a slight but significant increase in the percentage of Th1-type polyfunctional CD4^+^ T cells, which co-secrete IFN-γ, IL-2 and TNF-α and are indicators of vaccine-induced immunity against Tb (Figures 5B,D). Concerning zymosan, the generation of IFN-γ, IL-2 and TNF-α-producing cells among CD44^+^ CD4^+^ T cells was significantly enhanced in hSIGN mice with respect to WT and unvaccinated controls and percentages of Th1-polyfunctional T cells were also significantly higher (Figures 5B,E). It is important to note that we obtained the strongest immune responses with zymosan. Taken together, these results suggest that DC targeting *via* αDC-SIGN:P25 in combination with CTA1-DD or zymosan induces immunity against *Mtb via* the generation of Th1-type cells and polyfunctional CD4^+^ T cells.

### αDC-SIGN:P25 Plus Zymosan Generates Hyperactivated CD4^+^ T Cells

**Figure 6:**
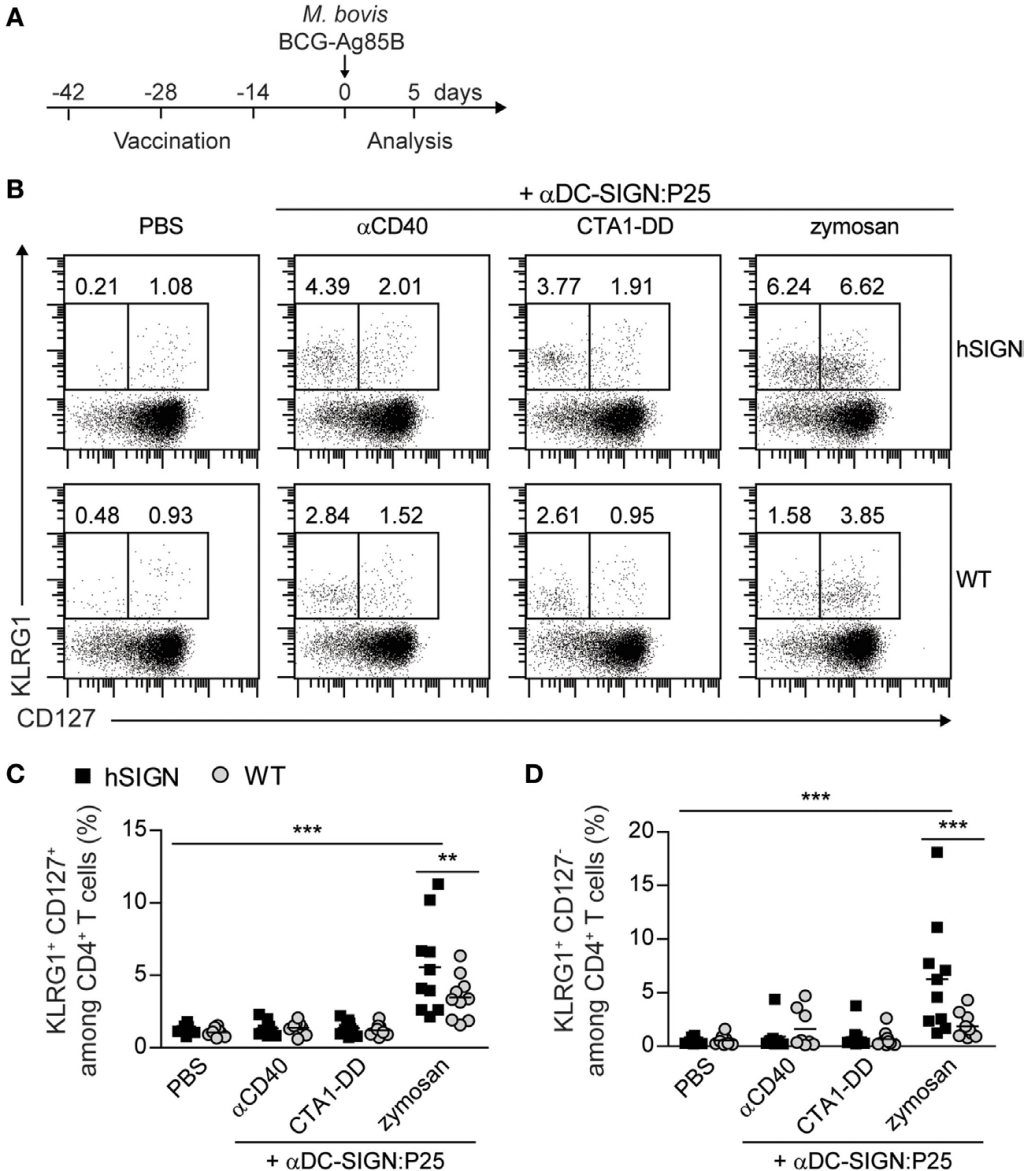
αDC-SIGN:P25 plus zymosan generates hyperactivated CD4^+^ T cells. **(A)** Experimental scheme. WT or hSIGN mice were vaccinated i.p. at days −42, −28, and −14 with vehicle (PBS) or αDC-SIGN:P25 (10 µg) plus αCD40 (10 µg), CTA1-DD (10 µg) or zymosan (200 µg). At day 0, mice were challenged i.v. with 2 × 10^6^ *Mycobacterium bovis* BCG-Ag85B. Five days later, mice were sacrificed and blood was isolated and stained for killer cell lectin-like receptor G1 (KLRG1) and CD127 surface expression. **(B)** Representative flow cytometry plots depicting percentage of KLRG1^+^ CD127^+^ and KLRG1^+^ CD127^−^ among CD4^+^ T cells. **(C,D)** Graphs represent percent of **(C)** KLRG1^+^ CD127^+^ and **(D)** KLRG1^+^ CD127^−^ among CD4^+^ T cells of vaccinated mice. Each symbol represents an individual mouse and results are pooled from two experiments with 4–5 mice per group. **p < 0.01 and ***p < 0.001; two-way ANOVA with Bonferroni’s *post hoc* test.

In recent years, several reports have shown that the state of differentiation and polarization of Th1 cells is important to determine their ability to control *Mtb* infection. Indeed, it has been demonstrated that less-polarized CD4^+^ T cells are more beneficial in terms of long-term protection against this pathogen (46–49). Thus, we analyzed the CD4^+^ T-cell memory and activation profile generated by the αDC-SIGN:P25 antibody in combination with the different adjuvants according to the experimental scheme in Figure 6A. hSIGN mice vaccinated with αDC-SIGN:P25 plus zymosan showed a significant increase in the percentage of both effector memory (KLRG^+^ CD127^+^) (Figures 6B,C) and terminally differentiated effector (KLRG1^+^ CD127^−^) CD4^+^ T cells in the blood (Figures 6B,D). On the contrary, αCD40 and CTA1-DD did not induce an increase in those populations. These results correlate with the fact that zymosan proved to be the strongest stimulus for the activation of CD4^+^ T cells and indicate that this adjuvant induces a rapid response upon infection with a tendency toward a more differentiated phenotype.

## Discussion

The development of an effective vaccine against Tb remains an unresolved public health issue. Of the many vaccine candidates in the clinical trial pipeline, so far none has proven to provide protection against infection or sterilizing immunity. In this study, we explored the potential of DC targeting *via* human DC-SIGN as a novel vaccine strategy against Tb. Using anti-human-DC-SIGN antibodies conjugated to Ag85B and P25 we could demonstrate that the CLR DC-SIGN can be efficiently targeted in DCs which results in the proliferation and IFN-γ production of P25-specific CD4^+^ T cells *in vitro* and *in vivo*. We also demonstrated that the type of T-cell response elicited by the anti-DC-SIGN antibodies and its strength is influenced by the adjuvant system used for the delivery. Finally, we show that anti-mycobacterial immunity based on Th1 and polyfunctional CD4^+^ T cells is achieved in a vaccination protocol with the anti-DC-SIGN antibody conjugated to P25.

We here provide evidence that human DC-SIGN is a potent targeting receptor that has the ability of inducing strong immune responses. First, in our *in vitro* results we show that low amounts of anti-DC-SIGN antibodies (0.001–0.1 µg/mL) are enough to induce Ag presentation and secretion of IFN-γ. This represents an advantage to *ex vivo* DC vaccines, where not only higher amounts of protein are needed but also the generation of autologous DCs is time consuming and expensive. Second, we also demonstrate that the presence of human DC-SIGN on DCs is sufficient to promote Ag presentation and the induction of an immune response upon immunization with anti-DC-SIGN antibodies *in vivo*. Though some response can be observed in WT and isotype-treated mice, probably due to the binding of the antibodies to Fc receptors or other receptors in an unspecific manner, the response in hSIGN mice is much stronger with high percentages of IFN-γ-producing cells. This corresponds with the fact that the IgG1 murine AZN-D1 antibody used for our targeting purposes has been shown to induce fast clathrin-dependent internalization of DC-SIGN upon binding and to direct Ags to late endosomal compartments facilitating efficient Ag presentation (50). Third, we have previously demonstrated that conventional DCs are the main target of the anti-DC-SIGN antibodies in this kind of vaccination approach. hSIGN mice express the human DC-SIGN receptor under the control of the CD11c promoter. Though CD11c is not exclusively expressed on DCs, we have previously shown that the transgene is predominantly expressed on CD11c^high^ SiglecH^−^ conventional DCs and can be detected in spleen, lymph nodes and lungs of hSIGN mice *via* immunohistochemistry (29, 30). Furthermore, the complementarity determining regions of AZN-D1 were grafted onto a human IgG2/IgG4 composite antibody with the objective of generating a humanized antibody which was later conjugated to the model Ag keyhole limpet hemocyanin (KLH) resulting in 100-fold more efficient targeting of human DCs compared with *ex vivo* Ag loading (51). Hence, extrapolating DC-SIGN targeting into a human vaccine could be an achievable aim. In this regard, it would be interesting to evaluate which subsets of DCs are the main targets for this vaccination approach.

Our results demonstrate that DC-SIGN targeting can generate P25-specific Th1 and polyfunctional T cells without significantly inducing IL-10-producing T cells or expanding the Treg population. Protective immunity against *Mtb* is known to rely on IFN-γ-secreting Th1 cells. The fact that Th1 cells play a central role in protection against Tb is based on several factors: (i) mice lacking CD4^+^ T cells, IFN-γ and IL-12 signaling or T-bet are highly susceptible to infection (4); (ii) individuals with genetic deficiencies in IFN-γ and IL-12 signaling are unable to control mycobacterial infections (52); (iii) HIV patients co-infected with Tb have increased risk of developing active disease (53). Apart from Th1 cells, other potential correlates of protection have been identified in the mouse model of Tb. The frequency and quality of polyfunctional CD4^+^ T cells was shown to correlate with protective immunity when comparing five different Tb vaccine models (live-attenuated, subunit, viral vectored, plasmid DNA and combination vaccines) (44). In contrast, Tregs have been shown to negatively influence vaccine efficacy (10). In this respect, our targeting strategy has the added advantage of avoiding the induction of these types of counteractive immune responses.

T-helper 17 cells have also been shown to provide protection against Tb either by recruiting Th1 cells to the infected lung or even by IFN-γ-independent mechanisms (5, 7, 54). Zymosan is a strong Th17-inducer stimulus, used in different IL-17-dependent experimental models such as arthritis (55). Upon treatment with zymosan, BMDCs were shown to promote the differentiation of naïve T cells into IL-17-producing cells *in vitro* and to support the induction of experimental autoimmune encephalomyelitis (EAE) symptoms in mice (40). In contrast, CTA1-DD was designed as a mucosal adjuvant capable of inducing powerful antibody responses (38). We demonstrated that both of these adjuvants were able to induce the activation of BMDCs and the secretion of IL-6, IL-23, and IL-1β, all important cytokines necessary for the induction of Th17 responses (56, 57). The fact that we could not observe differences in the percentages of P25-specific Th17 cells after vaccination could be due to the low amounts of IL-17A-producing cells generally observed in our experimental system. Likewise, in our previous reports *M. bovis* BCG-infected mice showed only marginal Th17 percentages at 21 days post-infection (58). This suggests that in our settings this T-cell subset is underrepresented, probably due to the restricted flora in our animal facility (SPF conditions). In this sense, there is evidence that the diversity of the intestinal microbiota and the presence of specific segmented filamentous bacteria can influence the amount of intestinal Th17 cells in mice from the same strain but from different facilities (59, 60). Thus, testing our vaccination system in mice with a more diverse microbiota would be important to define the importance of Th17 for anti-DC-SIGN-mediated immunity.

Several studies suggest that the activation status as well as the migration capacity of Th1 cells themselves is important to generate long-term protection. Distinct phenotypes based on the expression of programmed death-1 (PD-1) and killer cell lectin-like receptor G1 (KLRG1) were described among CD4^+^ T cells during *Mtb* infection. Thus, PD-1^+^ CD4^+^ T cells exhibit superior proliferation capacity though produce lower levels of IFN-γ, while KLRG1^+^ CD4^+^ T cells secrete high amounts of IFN-γ but show a terminally differentiated phenotype with reduced ability to proliferate (46). These KLRG1^+^ T cells express CX3CR1, are mainly found in the vasculature, and have diminished capacity to migrate into the lung parenchyma in contrast to KLRG1^−^ PD-1^+^ CXCR3^+^ cells which are mainly found in this tissue (48). As a consequence of their less-differentiated profile, KLRG1^−^ cells are able to persist and maintain anti-mycobacterial immunity after *Mtb* infection (47, 48, 61). In our experimental approach, the combination of the anti-DC-SIGN antibody conjugated to P25 with zymosan resulted in the strongest immune response in terms of cytokine production and also showed increased percentages of total KLRG1^+^ CD4^+^ T cells 5 days post-infection. These results suggest that zymosan induces a rapid increase of terminally activated cells but whether this is consequence of the vaccination or the ongoing infection remains unclear. Also, whether zymosan or CTA1-DD can generate Ag-specific KLRG1^−^ CD4^+^ T cells with the ability to persist in the long term is yet to be evaluated. Therefore, it would be interesting to assess the memory profile of the P25-specific CD4^+^ T cells generated upon vaccination with anti-DC-SIGN antibodies previous to challenge.

In spite of generating P25-specific CD4^+^ T cells against *Mtb*, we were unable to detect differences in the bacterial burden between unvaccinated and vaccinated mice. In our approach, we challenged the immunized mice with Ag85B-overexpressing BCG strain as a mycobacterial infection model. The rational behind this choice was based on the fact that Ag85B is expressed at lower levels in BCG compared to *Mtb*, where this protein is one of the major components of the bacterial culture filtrate (62). However, this model has a limitation in the sense that it is cleared much faster than the reference BCG Pasteur strain (data not shown). Hence, aerosol challenge with virulent *Mtb* would provide a better indication of whether DC-SIGN targeting has protective capacity against infection. In this sense, it would also be interesting to test whether the intranasal immunization route influences the type of response elicited by our anti-DC-SIGN antibodies. The mucosal administration of *M. bovis* BCG has shown to provide superior protection and favor the generation of lung-resident memory T cells (63, 64). Furthermore, upon intranasal administration of CTA1-DD in combination with QuilA-containing immune-stimulating complexes (ISCOMs) and the fusion protein Ag85B-ESAT-6, this adjuvant was shown to provide protection against Tb in the lung (38, 65, 66). Another advantage of the use of an antibody-based targeting strategy is the possibility of coupling different Ags to them. A broader range of *Mtb* epitopes can be covered in this manner, either CD4^+^ or CD8^+^, and therefore result in an improved immune response. CD8^+^ T-cell responses have also been shown to mediate protection through their ability to secrete high amounts of IFN-γ and TNF-α and kill *Mtb*-infected macrophages (67, 68). Thus, mucosal administration of anti-DC-SIGN antibodies conjugated to different CD4^+^ and CD8^+^ epitopes plus CTA1-DD or zymosan could support the long-term generation of *Mtb*-specific Th1, Th17 and CD8^+^ T cells along with other desirable memory T-helper subsets.

To conclude, we demonstrate here the potential of targeting human DC-SIGN to generate Ag-specific immune responses against *Mtb*. Even though further studies are needed in order to optimize this vaccination approach, we conclude that CLR targeting is a powerful tool that can be exploited to modulate immune responses and design novel vaccine strategies against global-health-threatening intracellular pathogens.

## Ethics Statement

This study was carried out in accordance with the recommendations of the German Animal Welfare Act. The protocol was approved by the Veterinary Institute of LAVES (Lower Saxony State Office for Consumer Protection and Food Safety, permit numbers: 12/0732 and 17/2472).

## Author Contributions

Conceptualization: LB and TS. Investigation: LV, PS, MG, MS, JB and MM. Resources: NL, DB, HL, HK and YK. Writing and visualization: LV, PS, TS and LB. Supervision and project administration: TS and LB. Funding acquisition: TS and LB.

## Conflict of Interest Statement

The authors declare that the research was conducted in the absence of any commercial or financial relationships that could be construed as a potential conflict of interest.

## Abbreviations

*Mtb, Mycobacterium tuberculosis*; Tb, tuberculosis; DCs, dendritic cells; CLR, C-type lectin receptor; Ag, antigen; DC-SIGN, DC-specific-ICAM3-grabbing-nonintegrin; BMDCs, bone-marrow-derived dendritic cells; GM-CSF, granulocyte-macrophage colony-stimulating factor; KLRG1, killer cell lectin-like receptor G1.
